# The Temporal Relationships and Associations between Cutaneous Manifestations and Inflammatory Bowel Disease: A Nationwide Population-Based Cohort Study

**DOI:** 10.3390/jcm10061311

**Published:** 2021-03-22

**Authors:** Yi-Teng Hung, Puo-Hsien Le, Chia-Jung Kuo, Yu-Chuan Tang, Meng-Jiun Chiou, Cheng-Tang Chiu, Chang-Fu Kuo, Yu-Huei Huang

**Affiliations:** 1 Department of Dermatology, Chang Gung Memorial Hospital, Linkou Branch, No. 5, Fuxing St., Guishan Dist., Taoyuan City 333, Taiwan; herovincent25@hotmail.com.tw; 2 School of Medicine, Chang Gung University, No. 259, Wenhua 1st Rd., Guishan Dist., Taoyuan City 333, Taiwan; puohsien@gmail.com (P.-H.L.); m7011@cgmh.org.tw (C.-J.K.); ctchiu0508@gmail.com (C.-T.C.); 3 Department of Gastroenterology, Chang Gung Memorial Hospital, Linkou Branch, No. 5, Fuxing St., Guishan Dist., Taoyuan City 333, Taiwan; 4 Center for Artificial Intelligence in Medicine, Chang Gung Memorial Hospital, Linkou Branch, No. 5, Fuxing St., Guishan Dist., Taoyuan City 333, Taiwan; tangyc41@gmail.com (Y.-C.T.); mengjun89@gmail.com (M.-J.C.); 5 Division of Rheumatology, Allergy and Immunology, Chang Gung Memorial Hospital, Linkou Branch, No. 5, Fuxing St., Guishan Dist., Taoyuan City 333, Taiwan

**Keywords:** association, epidemiology, inflammatory bowel disease, odds ratio, skin diseases, temporal relationship

## Abstract

The temporal relationships between inflammatory bowel disease (IBD)-associated cutaneous manifestations and IBD remain uncertain, with existing evidence mostly from separate cross-sectional studies. We sought to determine the risks of IBD-related dermatologic diseases before and after the diagnosis of IBD. We identified 2847 cases of IBD and 14,235 matched controls from the Taiwan National Health Insurance Research Database between 2003 and 2014. The risks of cutaneous manifestations before and after the diagnosis of IBD were estimated with multivariable-adjusted analyses. At diagnosis, IBD was associated with atopic dermatitis (odds ratio (OR) = 1.61; 95% confidence interval (CI), 1.14–2.28), erythema nodosum (OR = 7.44; 95%CI, 3.75–14.77), aphthous stomatitis (OR = 2.01; 95%CI, 1.72–2.35), polyarteritis nodosa (OR = 5.67; 95%CI, 2.69–11.98), rosacea (OR = 1.67, 95%CI = 1.19–2.35), and cutaneous T cell lymphoma (OR = 21.27; 95%CI, 2.37–191.00). IBD was associated with the subsequent development of pyoderma gangrenosum (hazard ratio (HR) = 17.79; 95%CI, 6.35–49.86), erythema nodosum (HR = 6.54; 95%CI, 2.83–15.13), polyarteritis nodosa (HR = 2.69; 95%CI, 1.05–6.90), hidradenitis suppurativa (HR = 2.48; 95%CI, 1.03–5.97), psoriasis (HR = 2.19; 95%CI, 1.27–3.79), rosacea (HR = 1.92; 95%CI, 1.39–2.65), and aphthous stomatitis (HR = 1.45; 95%CI, 1.22–1.72). This study clarified the associations and temporal relationships between cutaneous manifestations and IBD, highlighting the need for interdisciplinary care in the patient with specific dermatologic diseases presenting with abdominal symptoms, or the IBD patients with cutaneous lesions.

## 1. Introduction

Inflammatory bowel disease (IBD), divided into ulcerative colitis (UC) and Crohn’s disease (CD), is a chronic inflammatory disease of the intestinal tract and more prevalent in Caucasians than in Asians. However, recent studies have shown an increased incidence of IBD among the Chinese population [1]. IBD can affect organ systems other than the gastrointestinal tract. Extra-intestinal manifestations (EIMs) including involvements of the joints, eyes, skin, liver, and neurological and cardiovascular systems occur in 6–50% of IBD patients [2,3]. The prevalence of EIMs increases during the follow-up period [4], and 25% of EIMs occur before the onset of IBD [5]. Among EIMs, cutaneous manifestations are easier to be perceived by physicians, and their association with a more severe disease phenotype has been identified [6]. Therefore, it is crucial to recognize cutaneous EIMs in order to initiate appropriate treatments.

Cutaneous disorders, occurring in approximately 15% of IBD patients according to previous studies [2,5], may be divided into four categories: specific manifestations, reactive manifestations, associated disorders, and treatment-related cutaneous diseases [2,5]. Specific cutaneous manifestations reveal the histological findings identical to those of the intestinal lesions of IBD, such as metastatic Crohn’s disease [2,5]. Reactive cutaneous manifestations of IBD with immunological mechanisms triggered by common antigens shared by gut bacteria and skin include pyoderma gangrenosum (PG), erythema nodosum (EN), Sweet’s syndrome, and aphthous stomatitis [2,5]. Cutaneous disorders associated with IBD are reported in the patients of IBD without specific IBD-related histological features or mechanisms, including hidradenitis suppurativa (HS), atopic dermatitis (AD), rosacea, vitiligo, cutaneous polyarteritis nodosa (C-PAN), and psoriasis [2,7,8,9,10,11,12].

More than one EIM tends to occur in IBD patients in chronological order, and some cutaneous manifestations are significantly associated with other EIMs (arthritis, uveitis/iritis, and ankylosing spondylitis) [6]. IBD patients with skin manifestations may present with higher disease activity, complications, and comorbidities [6]. Furthermore, EN and aphthous stomatitis parallel underlying intestinal disease activity, suggesting the importance of skin manifestations in IBD [5,13,14,15]. This evidence shows the importance of being aware that the skin manifestations in IBD may be an indication of disease activity and could decrease the diagnostic delay of other EIMs or IBD.

So far, studies regarding skin manifestations in patients with IBD are scarce. Existing evidence of skin manifestations associated with IBD is predominantly derived from cross-sectional studies that mainly focus on the prevalence of EIMs, whereas the temporal relationships between cutaneous EIMs and IBD are not well understood. Therefore, we conducted this nationwide population-based study to determine the risks of cutaneous disorders in patients with IBD compared with matched controls at the time of the initial diagnosis of IBD and during the follow-up period.

## 2. Materials and Methods

### 2.1. Data Sources

This study was approved by the Institutional Review Board of Chang Gung Memorial Hospital (202000920B1). We used the Taiwan National Health Insurance Research Database (NHIRD) containing 99% of the health data of Taiwan residents. This database collected detailed demographic information, medical diagnoses, results of examinations, and medications. The diagnosis codes in the NHI database have been validated in several studies [16].

### 2.2. Definitions of Inflammatory Bowel Disease and Study Patients

People with a new diagnosis of CD or UC from 2003 to 2014, who had no existing evidence of IBD before the index date, were recruited. The case definition of IBD was a person with a catastrophic illness certificate for CD or UC (International Classification of Diseases, Ninth Revision, Clinical Modification (ICD-9-CM) codes 555 and 556).

The holders of a catastrophic illness certificate are permitted to a waiver of medical copayment. Therefore, the diagnosis must be confirmed by commissioned expert panels after reviewing comprehensive clinical assessments, laboratory tests, imaging studies, endoscopic findings, or pathologic reports before issuing the certificate to patients.

### 2.3. Matched Controls

Patients with IBD were matched with a 1:5 ratio to patients without IBD before the index date by sex and year of birth (±1 year) between 2003 and 2014. The index date was the initial diagnosis of IBD in cases, with the same date applied to their matched controls.

### 2.4. Comorbidities and Dermatologic Diseases

Demographic data including sex, age, biologics for IBD (adalimumab, infliximab, golimumab, and vedolizumab), and comorbidities (myocardial infarction, congestive heart failure, cerebrovascular disease, autoimmune disease, liver disease, diabetes mellitus (DM), renal disease, hypertension, and hyperlipidemia) were collected. The biologics targeting interleukin-17, 12, and 23 for the treatment of psoriasis or ankylosing spondylitis had not been reimbursed in Taiwan during the study period, and tofacitinib has not been approved yet. Therefore, these treatments were not included for analysis. The definitions for comorbidities and their corresponding ICD-9-CM and ICD-10-CM codes are listed in the Supplementary Materials (Supplementary Table S1). The diagnoses of cutaneous manifestations of IBD were identified by the ICD-9-CM and ICD-10-CM codes registered in the outpatient and hospitalization records in NHIRD (Supplementary Table S2). The skin manifestations associated with IBD in our analysis included psoriasis, vitiligo, AD (atopic dermatitis), PAN (polyarteritis nodosa), EN (erythema nodosum), aphthous stomatitis, PG, skin cancer (nonmelanoma skin cancer and melanoma), rosacea, HS, and cutaneous T cell lymphoma (CTCL). To ensure the accuracy of the diagnosis for psoriasis, AD, and vitiligo, we only included the cases that were given these diagnostic codes by dermatologists or rheumatologists at more than two outpatient visits or more than one hospitalization.

### 2.5. Study Period

The study patients and their matched controls were followed until the first skin manifestation, death, or the end of the study (31 December 2017). The dermatologic diseases before the index date were also collected in this study. The observation period in this study was from 1998 to 2017.

### 2.6. Statistical Analysis

The demographic characteristics for IBD patients and matched controls were compared with the chi-squared test for categorical variables. We calculated the prevalence of a specific dermatologic disease by dividing the number of people diagnosed with a particular disease before the index date by the number of people with IBD or matched controls. The odds ratio (OR) and 95% confidence interval (CI) were calculated by using a conditional logistic regression model to estimate the association between IBD and each coexisting skin manifestation. The incidence rates (IR) of cutaneous manifestations per 1000 person-years among IBD and non-IBD groups were estimated for at least three years after the index date. The hazard ratios (HRs) and 95% confidence interval (CI) were estimated by constructing three Cox proportional hazard models: model 1, unadjusted; model 2, adjusted for age, sex, and biologics use; and model 3, adjusted for covariates in model 2 and other comorbidities in demographic characteristics. The log rank test was used to estimate the cumulative probability of each dermatologic disease after the index date in subjects with IBD and controls. All analyses were performed by using SAS 9.4 (SAS Institute, Cary, NC, USA) with *p* < 0.05 identified to be statistically significant.

## 3. Results

### 3.1. Characteristics of Patients with IBD and Matched Controls

**Figure 1:**
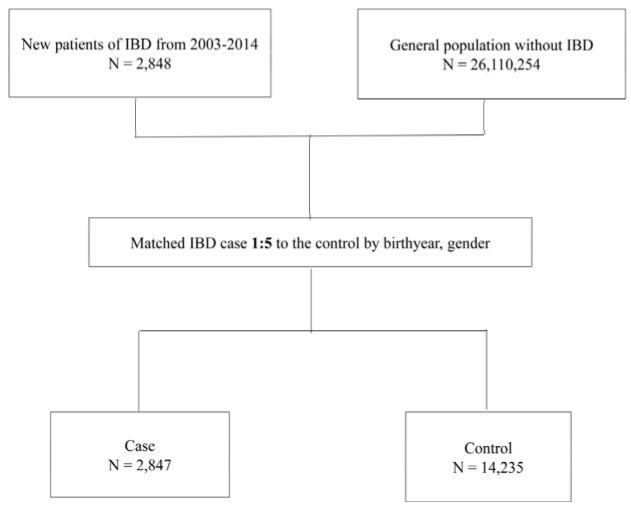
Flow chart of included patients for analyses.

After excluding one patient who was not matched successfully, 2847 patients with IBD and 14,235 age- and gender-matched controls were included in our analysis (Figure 1). Patient characteristics at diagnosis are listed in Table 1. The mean age was 40.54 ± 16.26 years, and males predominated in this cohort (*n* = 1798, 63.15%). People with IBD tended to have a higher prevalence of liver disease, DM, rheumatoid arthritis (RA), ankylosing spondylitis (AS), and other autoimmune diseases (excluding RA and AS). A higher proportion of patients with IBD used biologics.

### 3.2. Existing Skin Diseases at the Diagnosis of IBD

The ORs of certain dermatologic diseases calculated by the conditional logistic regression model before the index date of IBD were significantly higher in IBD patients compared with controls. These ORs (95% CIs) were 1.61 (1.14–2.28), 7.44 (3.75–14.77), 2.01 (1.72–2.35), 5.67 (2.69–11.98), 1.67 (1.19–2.35), and 21.27 (2.37–191.00) for AD, EN, aphthous stomatitis, PAN, rosacea, and CTCL, respectively (Table 2).

### 3.3. Risk of Developing Skin Disease after the Diagnosis of IBD

**Figure 2:**
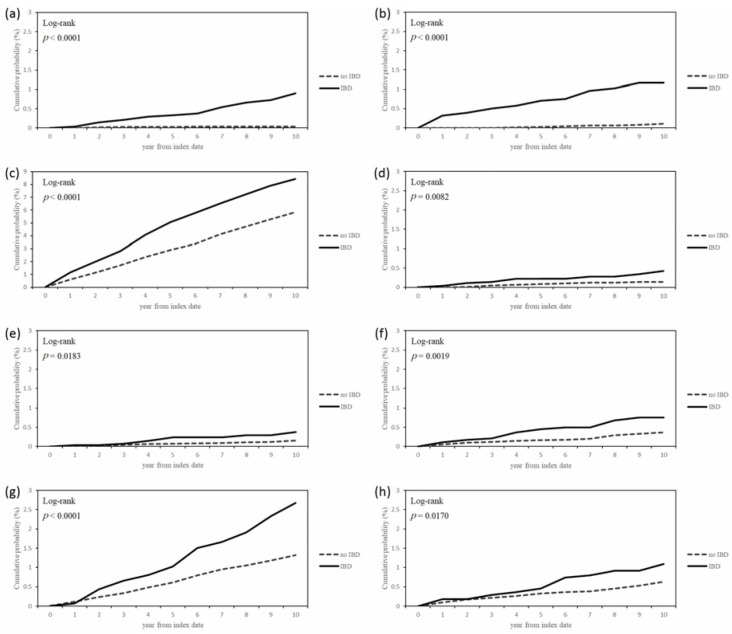
Cumulative probabilities of dermatologic diseases after index date: (**a**) pyoderma gangrenosum, (**b**) erythema nodosum, (**c**) aphthous stomatitis, (**d**) polyarteritis nodosa, (**e**) hidradenitis suppurativa, (**f**) psoriasis, (**g**) rosacea, and (**h**) atopic dermatitis.

After IBD diagnosis, the new diagnoses of PG, EN, PAN, HS, psoriasis, rosacea, and aphthous stomatitis in IBD patients were significantly higher compared with controls, and the corresponding adjusted HRs (95% CIs) were 17.79 (6.35–49.86), 6.54 (2.83–15.13), 2.69 (1.05–6.90), 2.48 (1.03–5.97), 2.19 (1.27–3.79), 1.92 (1.40–2.65), and 1.45 (1.22–1.72) (Table 3). The cumulative probabilities of developing certain dermatological diseases were also significantly higher in the IBD group compared to those in the control group (all with *p* < 0.05) (Figure 2).

### 3.4. Duration to Develop Skin Disease Following the Diagnosis of IBD

The median time to develop a cutaneous manifestation after the diagnosis of IBD ranged from 3.08 years for cutaneous T cell lymphoma to 5.67 years for PG. PAN and EN were among the earliest presentations of skin EIMs, with a median time of 3.15 and 3.55 years, respectively (Supplementary Table S3).

## 4. Discussion

To the best of our knowledge, this nationwide population-based study is the first study to investigate the temporal relationships between IBD and cutaneous manifestations and the risks of developing certain dermatologic diseases following IBD diagnosis. PG, EN, PAN, rosacea, and aphthous stomatitis were the cutaneous manifestations which significantly had a higher risk to exist at the time of initial diagnosis of IBD and to develop after the diagnosis; nevertheless, not all the cutaneous manifestations share a similar temporal relationship. The conspicuous differences in risks of an array of cutaneous diseases between subjects with IBD before their initial diagnosis and matched controls proposed that the aberrant immune response affects the skin earlier with obscure intestinal manifestations [5,17,18].

Reactive cutaneous manifestations discussed in this study include EN and PG, both of which are rare but extremely associated with IBD. EN is the most common cutaneous manifestation of IBD other than aphthous stomatitis, affecting 3–15% of IBD patients [19]. EN has been known to be correlated with the underlying disease activity and exacerbates with a flare-up of colitis [20]. We also noted a high incidence of EN (14.51 per 1000 person-years) among patients with IBD. A prospective cohort study found that EN rarely precedes the initial diagnosis of IBD [2,19,21]. However, a higher risk of pre-existing EN was noted in this study, which suggested that a long-term follow-up for patients with EN is warranted for early detection of IBD. PG is characterized by the appearance of pustules or papules that rapidly become deep excavating ulcers with undermined violaceous edges or vegetating lesions [15,22]. IBD often precedes the occurrence of PG and less than 15% of PG patients are diagnosed before the onset of IBD [23]. Our study concurs with this finding that most of the patients developed PG after the diagnosis of IBD with a significantly higher risk (HR = 17.79, 95% CI = 6.35–49.86) compared with controls. However, correlation of PG with IBD disease activity remains controversial and still needs more investigations [22,23].

Aphthous stomatitis may antedate, coexist with inflammatory bowel disease, or/and reflect the activity of intestine inflammation. In one systemic review featuring the oral manifestations of IBD, the prevalence of the specific manifestations and nonspecific manifestations (aphthous ulcerations) in IBD ranged from 0.7% to 37% in adults, and aphthous stomatitis was the most common among them, with 0.7% to 20% in adults [24]. Nonetheless, there was no chronological order mentioned in most existing studies. Our data showed 8.57% of IBD patients developed aphthae before initial diagnosis with a significantly higher OR (2.01, 95% CI = 1.72–2.35) compared with matched controls. The finding that more than half of the subjects developed aphthous stomatitis before IBD diagnosis in this study suggests that dermatologists play an important role in considering possible underlying IBD and in referring them for further examinations.

The relationship of psoriasis and IBD is believed to be both genetically and immunologically related [25]. Detection of IBD has been reported in psoriatic patients treated with interleukin-17 inhibitors, potentially impairing the function of the damaged epithelial barrier [26]. The significant bidirectional association between psoriasis and IBD was demonstrated in one systemic review and meta-analysis, with the prevalence of psoriasis in patients with CD and UC being 3.6% and 2.8%, respectively, and the prevalence of CD and UC in patients with psoriasis being 0.7% and 0.5%, respectively [8]. We observed an increased risk of psoriasis among patients with IBD following its diagnosis; on the contrary, psoriasis was not more prevalent before diagnosis compared with matched controls. It has been reported that patients with IBD who were treated with TNF-α inhibitors may develop paradoxical psoriasis [8,27]. After adjustment for biologics use, the significantly increased HR (2.19, 95% CI = 1.27–3.79) of psoriasis was still present among subjects with IBD during follow-up.

Rosacea and HS are chronic inflammatory skin diseases, which might share with IBD the commonalities in genetic susceptibility, dysregulation in innate and adaptive immunity, microbiota alteration, and trigger factors (smoking and obesity) [11,16,28,29,30,31]. The bidirectional association of IBD with rosacea or HS has been observed in previous reports and two prospective cohort studies revealed that subjects with rosacea or HS had a higher risk of developing IBD [9,12,29,32,33,34,35]. This study is, to the best of our knowledge, the first study to demonstrate that patients with IBD are more susceptible to develop rosacea and HS. Since rosacea and HS are both debilitating diseases, which have a great impact on patients’ quality of life, an early diagnosis and treatment intervention are imperative to prevent patients from suffering [7,36].

The most commonly reported cases of lymphoma associated with IBD were hepatosplenic T cell lymphoma and non-Hodgkin’s B cell lymphoma, which often developed following the use of TNF-α antagonists and immunosuppressants [37,38]. The case of CTCL was reported recently following use of infliximab and azathioprine for IBD [39]. Whether IBD alone is an independent risk factor for the development of lymphoma remains controversial [37,38]. We noted a higher risk of CTCL in subjects before the diagnosis of IBD and an increased cumulative probability of developing CTCL following the diagnosis compared with the matched controls. Even though the HR was not statistically significant, this finding raised our concern for the possible occurrence of CTCL in IBD patients.

Patients with IBD are at a higher risk for non-melanoma skin cancer (NMSC) and melanoma compared with non-IBD patients [40]. In one case–control study in the US, IBD patients had significantly elevated risks for NMSC (RR, 2.38; 95% CI, 1.99–2.85) and melanoma (RR, 2.85; 95% CI, 1.63–4.88) [41]. The prolonged use of thiopurine was demonstrated to increase the risk of NMSC, especially in combination with TNF antagonists [42,43,44,45]. Extremely rare cases of skin cancers were diagnosed in IBD patients in our study, and thus no significant result was obtained. This may be explained by the relatively lower incidence rate of skin cancers in Asians compared with Caucasians and some risk factors that cannot be acquired by NHIRD (family history, sun exposure, smoking, and HPV status). However, a population-based cohort study enrolling 2621 Chinese patients with IBD revealed that NMSC was significantly increased in both CD and UC [46].

AD is associated with IBD with common shared genetic, microorganism, and environmental factors [10,47,48,49]. The bidirectional association between IBD and AD was established in a previous report [49]. Patients with IBD had a significantly increased relative risk (RR) of 1.83 to have AD, and patients with AD compared with non-AD patients were 48% more likely to develop IBD [49]. A significant association with AD in the IBD group at index time is confirmed in our study, but no significantly elevated HR for AD was noted during follow-up.

A major strength of this study is the use of large representative population-based databases from NHIRD. This is the first study to investigate the temporal relationships between IBD and cutaneous manifestations and the risks of developing dermatologic diseases following IBD diagnosis. The types of dermatologic disease are also the most abundant and detailed among relevant studies. The results are not from clinical trials and otherwise reflect real-world clinical practice.

There are several limitations in this study. First, there may be some misclassifications of dermatologic diseases and comorbidities because some of them were diagnosed by physicians without established classification criteria, such as PG, AD, or rosacea. However, since specific diagnostic codes at more than one time point were necessary to be included in our study, the rate of misclassification should be very low. Second, ascertainment bias between people with IBD and the matched controls may exist. Third, residual confounding factors that cannot be acquired from NHIRD may exist and have an impact on the interpretation of our results. An example is smoking, which is associated with higher susceptibility to the development of EIMs [50]. Fourth, completeness of all IBD-related skin manifestations in this study was limited due to the rough definition in ICD-9-CM codes for some diseases, such as Sweet’s syndrome. PAN coded with ICD-9-CM 446.0 is not limited to cutaneous PAN. Rarely reported skin diseases in IBD (lichen planus, linear IgA bullous dermatosis, or epidermolysis bullosa acquisita) were not included [13]. Fifth, the severity of IBD cannot be ascertained from NHIRD, and thus the patients with early and subtle symptoms of IBD, not eligible for a catastrophic illness certificate (CIC), were not analyzed. The results may also not be generally applicable to populations with different ethnicities or geographic locations. Sixth, the effect of traditional immunosuppressants including systemic corticosteroids on the cutaneous diseases was not analyzed in this study. Indeed, de novo skin malignancies (especially squamous cell carcinoma) are more commonly seen in immunosuppressive patients than general populations. Finally, the number of patients with some skin diseases was too small to conduct statistically significant subgroup analyses by separating the patients into UC and CD. Hence, the differences in patients’ characteristics between those who developed each skin disorder before or after the diagnosis of IBD were also not investigated in the present study, but it is suggested that this is worthwhile further research in the future.

## 5. Conclusions

In conclusion, IBD patients are at a varying degree of risk of having certain cutaneous manifestations at or after the diagnosis of IBD. Recognizing the prevalent dermatologic manifestations occurring at the time of the diagnosis of IBD and the subsequent risk for them to develop after the diagnosis is important for clinical practice. This study suggests that gastrointestinal tract symptoms in patient with specific dermatologic diseases cannot be overlooked and detailed examination for a broad range of dermatologic diseases should be conducted for all patients with IBD. Interdisciplinary clinical care is extremely important for IBD.

## Figures and Tables

**Table 1:** Clinical characteristics of inflammatory bowel disease (IBD) patients and matched controls from 2003 to 2014.

Clinical Characteristics No. (%)	IBD (*n* = 2847)	No IBD (*n* = 14235)	*p*-Value *
Sex			
Male	1798 (63.15)	8990 (63.15)	1.00
Female	1049 (36.85)	5245 (36.85)	
Age, mean (SD), y	40.54 (16.26)	40.54 (16.27)	0.98
Age group, y			0.99
<20	272 (9.55)	1383 (9.72)	
20–39	1156 (40.60)	5748 (40.38)	
40–59	1072 (37.65)	5347 (37.56)	
≧60	347 (12.19)	1757 (12.34)	
IBD			
UC	2120 (74.46)	-	
CD	727 (25.54)	-	
Biologics use ^†^	471 (16.54)	6 (0.04)	<0.0001
Myocardial infarction	11 (0.39)	34 (0.24)	0.16
Congestive heart failure	31 (1.09)	136 (0.96)	0.51
Cerebrovascular disease	65 (2.28)	320 (2.25)	0.91
Rheumatoid arthritis	24 (0.84)	65 (0.46)	0.01
Ankylosing spondylitis	30 (1.05)	48 (0.34)	<0.0001
Autoimmune disease (without RA and AS)	22 (0.77)	67 (0.47)	0.04
Liver disease	69 (2.42)	180 (1.26)	<0.0001
Diabetes mellitus	131 (4.60)	819 (5.75)	0.01
Renal disease	17 (0.60)	98 (0.69)	0.59
Hypertension	348 (12.22)	1682 (11.82)	0.54
Hyperlipidemia	188 (6.60)	1041 (7.31)	0.18

IBD, inflammatory bowel disease; UC, ulcerative colitis; CD, Crohn’s disease; SD, standard deviation; RA, rheumatoid arthritis; AS, ankylosing spondylitis; y, years old. * *p*-Values were determined using a chi-squared test for categorical variables. ^†^ Biologics used in IBD included adalimumab, infliximab, golimumab, and vedolizumab.

**Table 2:** Pre-existing dermatologic disease at diagnosis of IBD.

	IBD (*n* = 2847) No. (%)	No IBD (*n* = 14235) No. (%)	Crude Odds Ratio (95% CI)	Adjusted Odds Ratio ^†^ (95% CI)^a^
**Reactive cutaneous manifestations**				
Pyoderma gangrenosum	2 (0.07)	0 (0.00)	-	-
Erythema nodosum	20 (0.70)	14 (0.10)	7.14(3.61–14.14)	7.44(3.75–14.77)
Aphthous stomatitis	244 (8.57)	640 (4.5)	2.02(1.73–2.36)	2.01(1.72–2.35)
**Associated cutaneous manifestations**				
Polyarteritis nodosa	15 (0.53)	13 (0.09)	5.77(2.75–12.12)	5.67(2.69–11.98)
Hidradenitis suppurativa	4 (0.14)	15 (0.11)	1.33(0.44–4.02)	1.16(0.38–3.52)
Psoriasis	14 (0.49)	69 (0.48)	1.01(0.57–1.81)	0.97(0.54–1.75)
Rosacea	45 (1.58)	139 (0.98)	1.63(1.16–2.29)	1.67(1.19–2.35)
Atopic dermatitis	43 (1.51)	135 (0.95)	1.60(1.13–2.26)	1.61(1.14–2.28)
Vitiligo	1 (0.04)	5 (0.04)	1.00(0.12–8.56)	1.19(0.14–10.27)
Skin cancer	5 (0.18)	16 (0.11)	1.56(0.57–4.27)	1.65(0.60–4.52)
Cutaneous T cell lymphoma	4 (0.14)	1 (0.01)	20.00(2.24–178.90)	21.27(2.37–191.00)

IBD, inflammatory bowel disease. ^†^ 95% confidence interval (CI) was calculated in a conditional logistic regression model after adjustment for age, sex, hypertension, heart disease, liver disease, chronic kidney disease, diabetes mellitus, hyperlipidemia, cerebrovascular disease, autoimmune disease (SLE, derma-tomyositis, systemic sclerosis, Sjogren’s syndrome, etc.), rheumatoid arthritis, and ankylosing spondylitis.

**Table 3:** Incidence and hazard ratio for dermatologic disease after diagnosis of IBD.

	IBD	No IBD
Reactive Cutaneous Manifestations		
**Pyoderma gangrenosum**		
Incidence/1000 person-years (95% CI)	9.12 (5.82–14.30)	0.48 (0.20–1.14)
Case Numbers No. (%)	19 (0.67)	5 (0.04)
Crude Hazard Ratio (95% CI)	19.32 (7.20–51.79)	1.00
Multivariable Hazard Ratio ^†^ (95% CI)	17.42 (6.26–48.47)	1.00
Multivariable Hazard Ratio ^‡^ (95% CI)	17.79 (6.35–49.86)	1.00
**Erythema nodosum**		
Incidence/1000 person-years (95%CI)	14.51 (10.15–20.76)	1.05 (0.58–1.89)
Case Numbers No. (%)	30 (1.06)	11 (0.08)
Crude Hazard Ratio (95% CI)	13.94 (6.98–27.82)	1.00
Multivariable Hazard Ratio ^†^ (95% CI)	6.78 (2.96–15.56)	1.00
Multivariable Hazard Ratio ^‡^ (95% CI)	6.54 (2.83–15.13)	1.00
**Aphthous stomatitis**		
Incidence/1000 person-years (95%CI)	106.20 (92.42–122.1)	73.18 (68.04–78.72)
Case Numbers No. (%)	198 (7.61)	723 (5.32)
Crude Hazard Ratio (95%CI)	1.47 (1.25–1.72)	1.00
Multivariable Hazard Ratio ^†^ (95% CI)	1.45 (1.22–1.72)	1.00
Multivariable Hazard Ratio ^‡^ (95% CI)	1.45 (1.22–1.72)	1.00
**Associated cutaneous manifestations**		
**Polyarteritis nodosa**		
Incidence/1000 person-years (95% CI)	4.34 (2.26–8.34)	1.52 (0.93–2.49)
Case Numbers No. (%)	9 (0.32)	16 (0.11)
Crude Hazard Ratio (95% CI)	2.86 (1.27–6.48)	1.00
Multivariable Hazard Ratio ^†^ (95% CI)	2.94 (1.24–6.95)	1.00
Multivariable Hazard Ratio ^‡^ (95% CI)	2.69 (1.05–6.90)	1.00
**Hidradenitis suppurativa**		
Incidence/1000 person-years (95%CI)	4.32 (2.25–8.30)	1.71 (1.08–2.72)
Case Numbers No. (%)	9 (0.32)	18 (0.13)
Crude Hazard Ratio (95% CI)	2.53 (1.14–5.64)	1.00
Multivariable Hazard Ratio ^†^ (95% CI)	2.44 (1.10–5.86)	1.00
Multivariable Hazard Ratio ^‡^ (95% CI)	2.48 (1.03–5.97)	1.00
**Psoriasis**		
Incidence/1000 person-years (95% CI)	9.65 (6.22–14.95)	4.31 (3.21–5.77)
Case Numbers No. (%)	20 (0.71)	45 (0.32)
Crude Hazard Ratio (95% CI)	2.26 (1.33–3.83)	1.00
Multivariable Hazard Ratio ^†^ (95% CI)	2.23 (1.27–3.89)	1.00
Multivariable Hazard Ratio ^‡^ (95% CI)	2.19 (1.27–3.79)	1.00
**Rosacea**		
Incidence/1000 person-years (95% CI)	29.85 (23.23–38.37)	15.22 (13.03–17.79)
Case Numbers No. (%)	61 (2.18)	158 (1.12)
Crude Hazard Ratio (95% CI)	1.98 (1.47–2.66)	1.00
Multivariable Hazard Ratio ^†^ (95% CI)	1.92 (1.40–2.64)	1.00
Multivariable Hazard Ratio ^‡^ (95% CI)	1.92 (1.39–2.65)	1.00
**Atopic dermatitis**		
Incidence/1000 person-years (95%CI)	11.70 (7.84–17.46)	6.74 (5.33–8.51)
Case Numbers No. (%)	24 (0.86)	70 (0.50)
Crude Hazard Ratio (95% CI)	1.75 (1.10–2.78)	1.00
Multivariable Hazard Ratio ^†^ (95% CI)	1.41 (0.80–2.51)	1.00
Multivariable Hazard Ratio ^‡^ (95% CI)	1.40 (0.80–2.46)	1.00
**Vitiligo**		
Incidence/1000 person-years (95%CI)	0	0.76 (0.38–1.52)
Case Numbers No. (%)	0 (0.00)	8 (0.06)
Crude Hazard Ratio (95% CI)	0	1.00
Multivariable Hazard Ratio ^†^ (95% CI)	0	1.00
Multivariable Hazard Ratio ^‡^ (95% CI)	0	1.00
**Skin cancer**		
Incidence/1000 person-years (95% CI)	0.96 (0.24–3.84)	2.00 (1.30–3.07)
Case Numbers No. (%)	2 (0.07)	21 (0.15)
Crude Hazard Ratio (95% CI)	0.48 (0.11–2.07)	1.00
Multivariable Hazard Ratio ^†^ (95% CI)	0.55 (0.13–2.36)	1.00
Multivariable Hazard Ratio ^‡^ (95% CI)	0.48 (0.12–2.03)	1.00
**Cutaneous T cell lymphoma**		
Incidence/1000 person-years (95% CI)	1.92 (0.72–5.11)	0.19 (0.05–0.76)
Case Numbers No. (%)	4 (0.14)	2 (0.01)
Crude Hazard Ratio (95% CI)	10.14 (1.86–55.31)	1.00
Multivariable Hazard Ratio ^†^ (95% CI)	5.93 (0.84–42.01)	1.00
Multivariable Hazard Ratio ^‡^ (95% CI)	6.18 (0.92–41.28)	1.00

IBD, inflammatory bowel disease. ^†^ 95% confidence interval (CI) was calculated in a Cox proportional hazard model after adjustment for age, sex, and biologics use. ^‡^ 95% confidence interval (CI) was calculated in a Cox proportional hazard model after adjustment for age, sex, biologics use, hypertension, heart disease, liver disease, chronic kidney disease, diabetes mellitus, hyperlipidemia, cerebrovascular disease, autoimmune disease (SLE, dermatomyositis, systemic sclerosis, Sjogren’s syndrome, etc.), rheumatoid arthritis, and ankylosing spondylitis.

## Data Availability

The data are based on the data from the National Health Insurance and the authors are not allowed to share the data. However, the data can be obtained by a reasonable request to the data holder, the Administration of the National Health Insurance.

## Supplementary Materials

The following are available online at https://www.mdpi.com/2077-0383/10/6/1311/s1, Table S1: ICD-9-CM and ICD-10-CM codes of comorbidities; Table S2: ICD-9-CM and ICD-10-CM codes of skin diseases; Table S3: Time (years) to develop skin diseases.

## Abbreviations

AD: atopic dermatitis; AS: ankylosing spondylitis; CD: Crohn’s disease; CI: confidence interval; CIC: catastrophic illness certificate; CTCL: cutaneous T cell lymphoma; DM: diabetes mellitus; EIM: extra-intestinal manifestation; EN: erythema nodosum; HS: hidradenitis suppurativa; HR: hazard ratio; IBD: inflammatory bowel disease; IR: incidence rate; NHIRD: National Health Insurance Research Database; NMSC: non-melanoma skin cancer; OR: odds ratio; PAN: polyarteritis nodosa; PG: pyoderma gangrenosum; RA: rheumatoid arthritis; TNF-α: tumor necrosis factor α; UC: ulcerative colitis.
