# Supplementary material for: The Temporal Relationships and Associations between Cutaneous Manifestations and Inflammatory Bowel Disease: A Nationwide Population-Based Cohort Study
Source: J Clin Med. 2021 Mar 22;10(6):1311. doi: 10.3390/jcm10061311 (PMC8004605; doi:10.3390/jcm10061311)
Supplement: Supplementary file 1 [file jcm-10-01311-s001.pdf]

Supplementary Table S1. ICD-9-CM and ICD-10-CM codes of comorbidities.

Comorbidity	ICD-9-CM	ICD-10-CM
Myocardial infarction	"410" "412"	"I21" "I22" "I25.2"
Congestive heart failure	"402.01" "402.11" "402.91" "404.01" "404.03" "404.11" "404.13" "404.91" "404.93" "429.3" "425" "428"	"I43" "I50" "I11.0" "I13.0" "I13.2" "I25.5" "I42.0" "I42.5" "I42.6" "I42.7" "I42.8" "I42.9" "P29.0"
Cerebrovascular disease	"362.34" "437" "438" "437.0" "437.1" "437.9" "781.4" "784.3" "997.0" "430" "431" "432" "433" "434" "435" "436" "381.2" "384.2"	"G45" "G46" "I60" "I61" "I62" "I63" "I64" "I65" "I66" "I67" "I68" "H34.0" "H34.1" "H34.2"
Ankylosing spondylitis (AS)	"720.0" "720.1" "720.2" "720.8" "720.9"	"M45.0-M45.9"
Rheumatoid arthritis (RA)	"714"	"M05" "M06"
Autoimmune disease (without AS and RA)	"710.0" "710.1" "710.2 " "710.3" "710.4" "710.5" "710.8" 710.9"	"M32" "M33" "M34" "M31.5" "M35.1" "M35.3" "M36.0"
Liver disease	"571.2" "571.5" "571.6" "571.8" "571.9" "456.0" "456.1" "456.2" "572.2" "572.3" "572.4"	"B18" "K73" "K74" "K28" "K70.0" "K70.1" "K70.2" "K70.3" "K70.9" "K71.3" "K71.4" "K71.5" "K71.7" "K76.0" "K76.2" "K76.3" "K76.4" "K76.8" "K76.9" "Z94.4" "I85.0" "I86.4" "K70.4" "K71.1" "K72.1" "K72.9" "K76.5" "K76.6" "K76.7"
Diabetes mellitus	"250.0" "250.1" "250.2" "250.3" "250.4" "250.5" "250.6" "250.7" "250.8" "250.9"	"E08" "E09" "E10" "E11" "E13"
Renal disease	"585" "586" "V56" "V42.0" "V45.1"	"N19" "Z49" "I12.0" "I13.2" "N18.5" "N18.6" "N25.0" "Z99.2" "I13.11"
Hypertension	"401" "402" "403" "404" "405"	"I10" "I11" "I12" "I13" "I15"
Hyperlipidemia	"272.0" "272.1" "272.2" "272.3" "272.4"	"E78"

Supplementary Table S2. ICD-9-CM and ICD-10-CM codes of skin diseases.

Skin disease	ICD-9-CM	ICD-10-CM
Psoriasis	"696.1"	"L40.0" "L40.1" "L40.2" "L40.3" L40.4" "L40.8" "L40.9"
Vitiligo	"709.01"	"L80"
Atopic dermatitis	"691.8"	"L20.9"
Polyarteritis nodosa	"446.0"	"M30.0"
Erythema nodosum	"695.2"	"L52"
Aphthous stomatitis	"528.2"	"K12.0"
Pyoderma gangre-nosum	"686.01"	"L88"
Skin cancer	"173.0-173.9" "172.0-172.9"	"C43.0-C43.9" "C44.0-C44.9"
Rosacea	"695.3"	"L71.0" "L71.1" "L71.8" "L71.9"
Hidradenitis suppu-rativa	"705.83"	"L73.2"
Cutaneous T cell lymphoma	"202.83"	"C84.A3"

Supplementary Table S3. Time (years) to develop skin diseases.

Skin disease	No.	Mean (y)	SD (y)	Median (y)	Min (y)	Max (y)
Psoriasis	20	5.05	3.68	4.34	0.25	12.72
Atopic dermatitis	24	4.77	3.26	4.93	0.36	12.87
Erythema nodosum	30	4.12	3.61	3.55	0.06	12.15
Aphthous stomatitis	198	4.62	3.36	3.90	0.03	14.30
Pyoderma gangrenosum	19	5.34	3.29	5.67	0.43	11.45
Polyarteritis nodosa	9	3.90	3.19	3.15	0.28	9.17
Rosacea	61	5.55	3.43	5.35	0.41	13.77
Hidradenitis suppurativa	9	5.59	4.08	4.89	0.64	13.86
Cutaneous T cell lymphoma	4	3.25	3.08	3.08	0.18	6.67

Abbreviation: SD, standard deviation; y, years; Min, minimum; Max, maximum.
